# 阿帕替尼联合CCI-779体外抑制小细胞肺癌细胞株NCI-H446的增殖和迁移

**DOI:** 10.3779/j.issn.1009-3419.2020.104.08

**Published:** 2020-04-20

**Authors:** 超 刘, 洪兵 张, 永文 李, 子禾 张, 睿峰 施, 松林 徐, 光胜 朱, 攀 王, 红雨 刘, 军 陈

**Affiliations:** 1 300052 天津，天津医科大学总医院肺部肿瘤外科 Department of Lung Cancer Surgery, Tianjin Medical University General Hospital, Tianjin 300052, China; 2 300052 天津，天津市肺癌研究所，天津市肺癌转移与肿瘤微环境重点实验室 Tianjin Key Laboratory of Lung Cancer Metastasis and Tumor Microenvironment, Tianjin Lung Cancer Institute, Tianjin Medical University General Hospital, Tianjin 300052, China

**Keywords:** 肺肿瘤, 阿帕替尼, mTOR抑制剂, CCI-779, 细胞周期, 凋亡, 细胞迁移, Lung neoplasms, Apatinib, mTOR inhibitor, CCI-779, Cell cycle, Apoptosis, Cell migration

## Abstract

**背景与目的:**

肺癌是世界上最常见的恶性肿瘤，其中小细胞肺癌是恶性程度最高的亚型，具有生长迅速、早期转移和高度血管化等特点。阿帕替尼（Apatinib）是我国自主研发的血管内皮生长因子受体2抑制剂，在多种实体瘤中疗效显著。本研究旨在探讨Apatinib对小细胞肺癌细胞株NCI-H446的体外作用以及联合哺乳动物雷帕霉素靶蛋白（mammalian target of rapamycin, mTOR）抑制剂CCI-779对小细胞肺癌的体外作用。

**方法:**

体外培养小细胞肺癌细胞株NCI-H446，CCK8法、细胞凋亡实验、细胞周期实验及Transwell实验检测Apatinib及联合mTOR抑制剂CCI-779对NCI-H446细胞增殖、凋亡、周期及迁移的影响；Western blot实验检测血管内皮生长因子受体和细胞周期相关蛋白的表达。

**结果:**

CCK8实验结果显示高浓度Apatinib能抑制NCI-H446细胞增殖；细胞凋亡实验结果显示高浓度Apatinib诱导NCI-H446细胞凋亡；Transwell实验结果显示高浓度Apatinib抑制NCI-H446细胞迁移；联合mTOR抑制剂CCI-779后，低浓度Apatinib便能抑制NCI-H446细胞增殖和迁移，诱导细胞凋亡。

**结论:**

Apatinib对小细胞肺癌细胞株NCI-H446的作用具有浓度依赖性特征，高浓度Apatinib能够抑制NCI-H446细胞增殖和迁移，诱导细胞凋亡，与mTOR抑制剂CCI-779联用能增加NCI-H446细胞对Apatinib的敏感性。

## Abstract (en)

**Background and objective:**

Lung cancer is the most common malignancy world-wide. Small cell lung cancer is the deadliest subtype of lung cancer, which features such as rapid growth, early metastasis, and high vascularization. Apatinib is a vascular endothelial growth factor receptor 2 inhibitor independently developed in China, which has a significant inhibition in a variety of solid tumors. The purpose of this study is to investigate the effects of Apatinib alone or Apatinib combined with mammalian target of rapamycin (mTOR) inhibitor, CCI-779, on small cell lung cancer cell line NCI-H446 *in vitro*.

**Methods:**

The small cell lung cancer cell line NCI-H446 was grew *in vitro*. The effects of Apatinib alone or Apatinib combined with CCI-779 on proliferation, apoptosis, cell cycle and migration of NCI-H446 small cell lung cancer cells were detected by CCK8; FACS and transwell assays were also carried out; Western blot assays were used to detect vascular endothelial growth factor and cell cycle related protein expression.

**Results:**

CCK8 assays showed that high concentration of Apatinib could inhibit the proliferation of NCI-H446 cells. Apoptosis assays showed that high concentration of Apatinib could induce NCI-H446 cell apoptosis. Transwell assays showed that high concentration of Apatinib could inhibit NCI-H446 cell migration. After combined with mTOR inhibitor CCI-779, low concentration of Apatinib could inhibit the proliferation and migration of NCI-H446 small cell lung cancer cells and induce apoptosis.

**Conclusion:**

Apatinib has a concentration-dependent effect on the small cell lung cancer cell line NCI-H446. High concentration of Apatinib can inhibit the proliferation and migration of NCI-H446 small cell lung cancer cells, induce apoptosis. Apatinib combined with the mTOR inhibitor CCI-779 can sensitize the NCI-H446 cells to Apatinib.

肺癌是最常见的癌症以及癌症相关死亡的主要原因，可分为非小细胞肺癌（non-small cell lung cancer, NSCLC）和小细胞肺癌（small cell lung cancer, SCLC）。其中SCLC约占所有肺癌的10%-15%^[1]^，是一种高侵袭、高致死率、易广泛转移的肺癌类型，早期即可出现血行转移，约60%患者确诊时已有远处转移^[2]^，5年生存率仅为7%^[3, 4]^。SCLC具有生长迅速、高度血管化的特点。血管生成是恶性肿瘤进展过程中最关键的过程之一，其为肿瘤的生长提供营养物质，促进肿瘤的生长，与肿瘤的发生、增殖和转移密切相关^[5]^。血管内皮生长因子（vascular endothelial growth factor, VEGF）是血管生成的重要调控分子，能够与血管内皮生长因子受体2（vascular endothelial growth factor receptor 2, VEGFR2）结合形成正反馈，调控血管内皮细胞的增殖、分化和迁移，促进新生血管形成。阻断VEGFR2可抑制肿瘤诱导的血管生成^[6]^。此外，几乎所有SCLC患者都存在*TP53*和*RB*基因失活或突变^[7, 8]^。*TP53*和*RB*基因是重要的抑癌基因，其突变会导致细胞周期调控异常，细胞持续增殖，促进肿瘤的发生及发展。

阿帕替尼（Apatinib）是一种小分子酪氨酸激酶抑制剂，能够选择性抑制VEGFR2的活性，从而抑制VEGF介导的细胞增殖、肿瘤微血管密度^[9]^。Apatinib已被证实在多种类型肿瘤中发挥作用，如胃癌、肝癌、乳腺癌、结直肠癌以及NSCLC^[10-12]^，但是Apatinib对SCLC细胞的作用尚需探索。CCI-779是一种哺乳动物雷帕霉素靶蛋白（mammalian target of rapamycin, mTOR）抑制剂，能够抑制肿瘤细胞增殖及VEGF产生，诱导细胞周期停滞在G_1_期，由于SCLC均存在细胞增殖异常及VEGF调控异常，mTOR抑制剂有望抑制其增殖。此外，联合mTOR周期抑制剂有可能增加SCLC对Apatinib的敏感性。本研究通过体外实验探讨Apatinib对SCLC细胞株的作用以及联合CCI-779能否增加SCLC细胞株对Apatinib敏感性。

## 1 材料与方法

### 1.1 细胞株和主要试剂

人SCLC细胞NCI-H446（*TP53*突变、*RB*突变）购自中国科学院细胞库（中国上海），阿帕替尼（Apatinib mesylate, S2221）、CCI-779（NSC683864, S1044）均购自Selleck公司。抗体：VEGFR2抗体购自Abcam公司，CDK4、CDK6、GAPDH抗体均购自Cell Signaling Technology公司。RPMI-1640培养基、胎牛血清（fetal bovine serum）、胰蛋白酶均购自GIBCO公司。CCK-8细胞增殖试剂盒、二甲基亚砜（dimethyl sulfoxide, DMSO）均购自碧云天公司。PI/RNase染色液、细胞凋亡试剂盒均购自BD公司。Transwell购自美国Corning公司。

### 1.2 细胞培养

将细胞从-80 ℃冰箱中取出，在37 ℃水浴锅中快速复温，1, 000 rpm离心3 min，弃掉上清液，用新鲜培养基重悬细胞沉淀，培养于含10%胎牛血清的RPMI-1640培养基，置于37 ℃、5%CO_2_饱和湿度的培养箱中。待细胞生长至90%左右融合度时，弃掉培养基，用PBS清洗1遍-2遍，0.25%胰酶-EDTA消化传代。所有实验均采用对数生长期细胞。

### 1.3 CCK8法测定细胞活力

收集呈对数期稳定生长的NCI-H446细胞，细胞计数板计数，调整细胞悬液浓度至2.5×10^4^/mL，向96孔板中每孔加入200 μL悬液，37 ℃、5%CO_2_培养过夜，待细胞贴壁后，设置Apatinib浓度梯度为0 μmol/L、1 μmol/L、2 μmol/L、4 μmol/L、8 μmol/L、16 μmol/L、32 μmol/L、64 μmol/L、128 μmol/L，每个浓度设4个复孔，每个复孔加入200 μL不同浓度的Apatinib。37 ℃、5%CO_2_孵育24 h后，弃掉培养基，每孔加入100 μL含10% CCK-8的完全培养基，避光，37 ℃培养箱中孵育1 h，用酶标仪测定在450 nm处的吸光度，药物抑制率计算公式：（对照组OD值-实验组OD值）/对照组OD值×100%，计算出药物的半数抑制浓度（half inhibitory concentration, IC_50_）。以同样的方法测定CCI-779对NCI-H446细胞的抑制率及IC_50_。

### 1.4 细胞周期实验

将NCI-H446细胞接种至6孔板中，加入完全培养基，待细胞汇合率达到70%-80%时，吸去孔内培养基，将各孔分为对照组、CCI-779处理组、低浓度Apatinib处理组、高浓度Apatinib处理组、低浓度Apatinib联合CCI-779处理组、高浓度Apatinib联合CCI-779处理组，各组分别加入不同浓度药物2 mL，37 ℃、5%CO_2_孵育24 h。收集1×10^6^个细胞于流式管中，用预冷的PBS清洗细胞，1, 000 rpm离心3 min，弃上清，逐滴加入1 mL-2 mL预冷的75%乙醇，涡旋混匀细胞，4 ℃避光过夜，2, 000 rpm离心细胞10 min，弃上清，PBS清洗细胞2次以去除乙醇，将细胞重悬于0.5 mL PI/RNase染色液，室温避光孵育15 min，1 h之内上流式细胞仪进行检测。

### 1.5 细胞凋亡实验

将NCI-H446细胞接种至6孔板中，加入完全培养基，待细胞汇合率达到70%-80%时，吸去孔内培养基，将各孔分为对照组、CCI-779处理组、低浓度Apatinib处理组、高浓度Apatinib处理组、低浓度Apatinib联合CCI-779处理组、高浓度Apatinib联合CCI-779处理组，各组分别加入不同浓度药物2 mL，37 ℃、5%CO_2_孵育24 h。收集5×10^5^个细胞于流式管中，用预冷的PBS清洗细胞，1, 000 rpm离心3 min，弃上清，加入100 μL 1×Binding Buffer重悬细胞，加入5 μL Annexin V-FITC，37 ℃培养箱中孵育10 min；加入5 μL PI Staining Solution，轻轻混匀，避光、室温反应10 min；加入400 μL 1×Binding Buffer，混匀，样品在1 h内用流式细胞仪检测。

### 1.6 Transwell迁移实验

将NCI-H446细胞接种至6孔板中，加入完全培养基，待细胞汇合率达到70%-80%时，吸去孔内培养基，将各孔分为对照组、CCI-779处理组、低浓度Apatinib处理组、高浓度Apatinib处理组、低浓度Apatinib联合CCI-779处理组、高浓度Apatinib联合CCI-779处理组，各组分别加入不同浓度药物2 mL，37 ℃、5%CO_2_孵育24 h。使用无血清RPMI-1640培养基调整各组细胞浓度为1×10^5^/mL，分别取200 μL加入上室，于下室加入600 μL含10%胎牛血清的RPMI-1640培养基，置于37 ℃、5%CO_2_培养箱，培养24 h。取出小室后，用棉签轻柔拭去上室细胞，4%多聚甲醛固定15 min，结晶紫室温染色25 min，并用PBS将多余染色液洗净，显微镜下观察穿过的细胞，每个样本随机选取5个视野进行拍照计数，计算平均值。

### 1.7 蛋白免疫印迹实验

将NCI-H446细胞接种至6孔板中，当细胞融合度达到70%-80%时，加入不同浓度药物处理，24 h后提取蛋白，BCA法测定蛋白浓度。加入SDS-PAGE蛋白上样缓冲液，充分混匀，100 ℃金属浴加热15 min。每种样品取30 μg上样电泳，湿转法电转移至PVDF膜，含5%脱脂牛奶的TBST摇床上室温封闭2 h。按照不同分子量剪切PVDF膜，加入对应的抗体，4 ℃摇床孵育过夜。次日用TBST室温清洗3次，每次5 min，加HRP标记的二抗，室温孵育1 h，显色曝光，分析条带灰度值。

### 1.8 统计学方法

应用Graphpad Prism 8.0软件对数据进行统计学分析并作图，组间数据比较采用单因素*t*检验，*P*值取双侧检验，*P* < 0.05为有统计学差异。

## 2 结果

### 2.1 高浓度Apatinib抑制NCI-H446细胞增殖

Apatinib按照0 μmol/L、1 μmol/L、2 μmol/L、4 μmol/L、8 μmol/L、16 μmol/L、32 μmol/L、64 μmol/L、128 μmol/L的浓度梯度处理NCI-H446细胞24 h后，CCK8法计算出其IC_50_为30.44 μmol/L。如图 1所示，当药物浓度 < 10 μmol/L时，Apatinib对NCI-H446细胞活力几乎没有影响；当药物浓度 > 10 μmol/L时，Apatinib开始逐渐抑制NCI-H446细胞增殖，药物浓度 > 30.44 μmol/L后才能将NCI-H446细胞活力抑制在50%以下，提示Apatinib对NCI-H446的作用具有浓度依赖性，高浓度Apatinib才能明显抑制NCI-H446细胞增殖。

**1:**
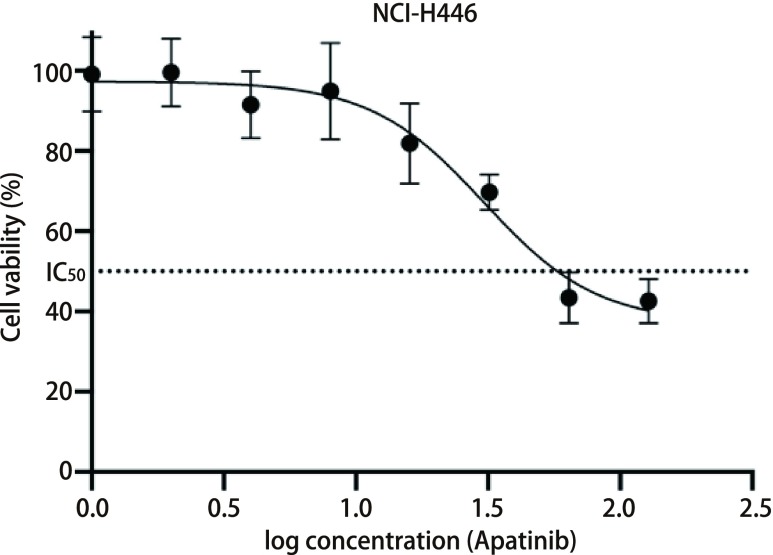
高浓度Apatinib抑制NCI-H446细胞增殖。不同浓度的Apatinib处理NCI-H446细胞24 h后，CCK8法计算出其IC_50_为30.44 μmol/L。当药物浓度 > 10 μmol/L时，Apatinib开始逐渐抑制NCI-H446细胞增殖，药物浓度 > 30.44 μmol/L后才能将NCI-H446细胞活力抑制在50%以下。 High concentration of Apatinib inhibits NCI-H446 small cell lung cancer cells proliferation. After the NCI-H446 small cell lung cancer cells were treated with Apatinib at different concentrations for 24 h, the IC_50_ calculated by the CCK8 was 30.44 μmol/L. When the drug concentration > 10 μmol/L, Apatinib gradually inhibits the proliferation of NCI-H446 small cell lung cancer cells, and the NCI-H446 small cell lung cancer cells viability can be suppressed below 50% when concentration > 30.44 μmol/L. IC_50_: half inhibitory concentration.

### 2.2 高浓度Apatinib诱导NCI-H446细胞凋亡

为了研究Apatinib对NCI-H446细胞凋亡的影响，我们分别用低浓度（12 μmol/L、16 μmol/L）和高浓度（28 μmol/L、32 μmol/L）Apatinib处理NCI-H446细胞24 h，流式细胞仪检测细胞凋亡情况。如图 2所示，对照组凋亡细胞占总细胞的比例为4.18%±0.30%，低浓度Apatinib处理组分别为4.29%±0.25%、4.88%±0.17%，高浓度Apatinib处理组分别为10.09%±0.37%、11.22%±0.81%。与对照组相比，低浓度Apatinib处理组NCI-H446细胞凋亡没有显著变化（*P* > 0.5），高浓度Apatinib处理组NCI-H446细胞凋亡明显增加（*P* < 0.001），提示高浓度Apatinib能诱导NCI-H446细胞凋亡。

**2:**
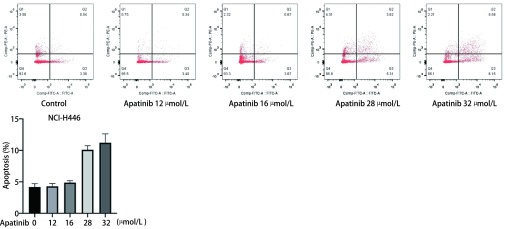
高浓度Apatinib诱导NCI-H446细胞凋亡。与对照组相比，低浓度Apatinib处理组NCI-H446细胞凋亡没有显著变化（*P* > 0.5），高浓度Apatinib处理组细胞凋亡明显增加（*P* < 0.000, 1）。 High concentration of Apatinib induces apoptosis in NCI-H446 small cell lung cancer cells. Compared with the control group, low concentration of Apatinib didn't induce apoptosis of NCI-H446 small cell lung cancer cells (*P* > 0.5), while the high concentration of Apatinib significantly increased the apoptosis of NCI-H446 small cell lung cancer cells (*P* < 0.000, 1).

### 2.3 高浓度Apatinib抑制NCI-H446细胞迁移

为了研究Apatinib对NCI-H446细胞迁移的影响，我们分别用低浓度（12 μmol/L、16 μmol/L）和高浓度（28 μmol/L、32 μmol/L）Apatinib处理NCI-H446细胞24 h，将细胞加入Transwell小室中，24 h后观察细胞迁移数目。如图 3所示，对照组细胞迁移数目为（271.60±9.60）个，低浓度Apatinib处理组分别为（285.60±7.61）个、（255.80±3.60）个，高浓度Apatinib处理组分别为（78.20±10.30）个、（11.80±2.60）个。与对照组相比，低浓度Apatinib处理组NCI-H446细胞迁移无显著变化（*P* > 0.5），高浓度Apatinib处理组显著抑制NCI-H446细胞迁移（*P* < 0.000, 1），提示高浓度Apatinib显著抑制NCI-H446细胞迁移。

**3:**
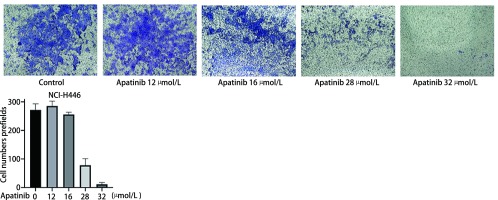
高浓度Apatinib抑制NCI-H446细胞迁移。与对照组相比，低浓度Apatinib处理组NCI-H446细胞迁移无显著变化（*P* > 0.5），高浓度Apatinib处理组显著抑制NCI-H446细胞迁移（*P* < 0.000, 1）。 High concentration of Apatinib inhibits the migration of NCI-H446 small cell lung cancer cells. Compared with the control group, low concentration of Apatinib didn't inhibit the migration of NCI-H446 small cell lung cancer cells (*P* > 0.5), while the high concentration of Apatinib significantly inhibited the migration of NCI-H446 small cell lung cancer cells (*P* < 0.000, 1).

### 2.4 Apatinib联合CCI-779增加NCI-H446细胞对Apatinib的敏感性

如图 4A所示，CCI-779按照0 μmol/L、1 μmol/L、2 μmol/L、4 μmol/L、8 μmol/L、16 μmol/L、32 μmol/L、64 μmol/L、128 μmol/L的浓度梯度处理NCI-H446细胞24 h后，CCK8法计算出其IC_50_为13.59 μmol/L，提示CCI-779能抑制NCI-H446活性。

**4:**
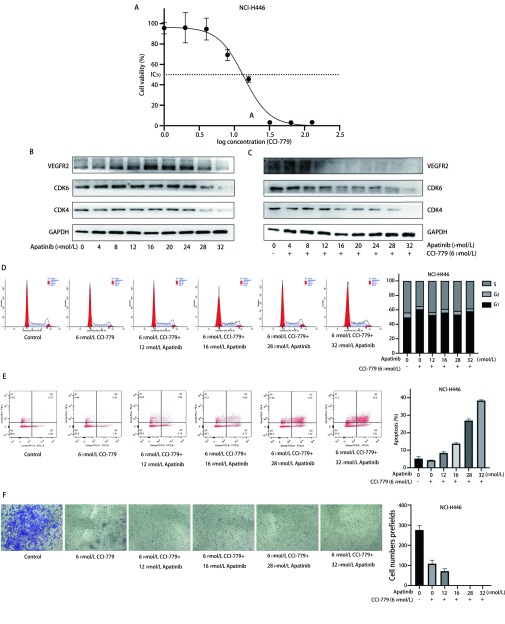
Apatinib联合CCI-779抑制NCI-H446细胞细胞周期进展及细胞迁移 Effect of Apatinib combined with CCI-779 inhibits cell cycle and migration of NCI-H446 small cell lung cancer cells

首先，选取药物浓度为6 μmol/L（1/2 IC_50_）的CCI-779与Apatinib联用。在Apatinib 0 μmol/L、4 μmol/L、8 μmol/L、12 μmol/L、16 μmol/L、20 μmol/L、24 μmol/L、28 μmol/L、32 μmol/L浓度梯度下，应用蛋白免疫印迹实验，观察单药Apatinib和Apatinib联合CCI-779时细胞周期蛋白的变化。如图 4B-4C所示，单药Apatinib处理NCI-H446细胞时，Apatinib浓度 > 24 µmol/L，细胞周期蛋白CDK4、CDK6蛋白表达才出现明显降低，VEGFR2才被明显抑制，进一步提示Apatinib的作用具有浓度依赖性，较高浓度才能抑制VEGFR2的表达；Apatinib联合CCI-779处理NCI-H446细胞时，Apatinib在较低浓度就能降低CDK4、CDK6蛋白表达，明显抑制VEGFR2，提示CCI-779联合Apatinib能够降低细胞周期蛋白的表达，增加Apatinib对VEGFR2的抑制作用。

为了进一步研究Apatinib联合CCI-779对NCI-H446细胞的作用，我们将实验分为对照组、CCI-779处理组、低浓度Apatinib（12 μmol/L、16 μmol/L）联合CCI-779处理组、高浓度Apatinib（28 μmol/L、32 μmol/L）联合CCI-779处理组，分别进行细胞周期实验、细胞凋亡实验以及细胞迁移实验。如图 4D所示，与对照组相比，CCI-779处理组、低浓度Apatinib联合CCI-779处理组、高浓度Apatinib联合CCI-779处理组均出现明显的G_1_期阻滞（*P* < 0.05）。如图 4E所示，对照组凋亡细胞占总细胞的比例为5.20%±0.65%，CCI-779处理组为4.26%±0.08%，低浓度Apatinib联合CCI-779处理组分别为8.41%±0.43%、13.76%±0.26%，高浓度Apatinib联合CCI-779处理组分别为29.96%±0.56%、38.34%±0.31%。与对照组相比，CCI-779处理组NCI-H446细胞凋亡无显著变化（*P* > 0.5），低浓度和高浓度Apatinib联合CCI-779处理组NCI-H446细胞凋亡明显增加（*P* < 0.000, 1）。如图 4F所示，对照组细胞迁移个数为（275.60±9.72）个，CCI-779处理组为（108.40±7.83）个，低浓度Apatinib联合CCI-779处理组分别为（71.30±5.33）个和0个，高浓度Apatinib联合CCI-779处理组迁移到下室细胞数为0。与对照组相比，CCI-779处理组、低浓度Apatinib联合CCI-779处理组和高浓度Apatinib联合CCI-779处理组显著抑制NCI-H446细胞迁移（*P* < 0.000, 1）。上述结果表明，Apatinib联合CCI-779能够增加NCI-H446细胞对Apatinib的敏感性，抑制NCI-H446细胞的增殖和迁移，导致细胞周期G_1_期阻滞，并诱导细胞凋亡。

## 3 讨论

SCLC是一种高侵袭、高致死率、易广泛转移的肺癌类型，大部分患者确诊时即已发生转移，可进行手术切除的SCLC患者比例低，仅有约5%的患者能够早期发现并完成手术治疗，而且手术治疗仅适用于临床分期I期（T1-2N0），并且纵隔淋巴结未被侵犯的局限期SCLC患者^[13]^。大部分患者需接受药物治疗，SCLC患者对化疗和放疗敏感，但易于复发并且出现耐药性，局限期患者化疗联合放疗后，5年生存率不到15%，而广泛期患者的2年生存率不到5%^[14]^，探索新的抗肿瘤药物成为提高SCLC疗效的重要途径之一。精准医学的发展促进了对肿瘤发生发展机制和分子生物学的理解，靶向治疗为NSCLC的治疗策略带来了翻天覆地的变化^[15]^，与此同时，对SCLC基因组学的研究也成为了当下的热点。VEGF信号通路中关键因子如VEGF、低氧诱导因子-1α（hypoxia inducible factor-1α, HIF-1α）、VEGFR等在SCLC中均呈过表达状态, 与肿瘤细胞的增殖、转移、侵袭以及预后不良密切相关^[16, 17]^。研究^[18]^表明，VEGF相关信号通路能够促进SCLC的生长及转移，且SCLC具有高度血管化的特点，提示靶向VEGF信号通路的药物可能改善SCLC的疗效。血管生成能够促进新生异常血管网的产生，改变肿瘤微环境，在肿瘤细胞的转移和生长过程中发挥着重要作用^[19]^。VEGF信号通路是血管生成的重要组成部分，靶向VEGF信号通路的抗血管生成药物可以抑制肿瘤新生血管生成，使现有的肿瘤血管退化，阻止肿瘤细胞摄取营养物质，从而持续抑制肿瘤细胞的生长和转移，最终达到抑制肿瘤生长的作用。

Apatinib是一种选择性抑制VEGFR-2的小分子酪氨酸激酶抑制剂，Apatinib在多种恶性肿瘤中疗效显著，但在SCLC中研究较少。Apatinib在三线及三线以上的SCLC治疗中有一定的效果。本研究通过体外试验进一步证实证实，Apatinib对SCLC细胞株NCI-H446的作用具有浓度依赖性特征，高浓度Apatinib能够抑制NCI-H446细胞增殖和迁移，诱导细胞凋亡，且与mTOR抑制剂CCI-779联用能够提高NCI-H446细胞对Apatinib的敏感性，在较低浓度时便能抑制NCI-H446细胞增殖和迁移，导致细胞周期G_1_期阻滞，并诱导细胞凋亡。
